# Impacts of risk-stratified inpatient penicillin allergy label delabeling on subsequent antimicrobial spectrum index and costs

**DOI:** 10.1017/ash.2024.421

**Published:** 2024-10-03

**Authors:** Milner Staub, George E. Nelson, Kelly Byrge, Grace Koo, Whitney J. Nesbitt, Joanna L. Stollings, Minhua Zhang, Cosby A. Stone

**Affiliations:** 1 Division of Infectious Diseases, Department of Medicine, Vanderbilt University Medical Center, Nashville, TN, USA; 2 Medicine Service Line, Infectious Diseases Section, Veterans Affairs Tennessee Valley Healthcare System, Nashville, TN, USA; 3 Division of Allergy and Immunology, Department of Medicine, Vanderbilt University Medical Center, Nashville, TN, USA; 4 Department of Pharmaceutical Services, Vanderbilt University Medical Center, Nashville, TN, USA; 5 Quality, Safety, and Risk Prevention, Vanderbilt University Medical Center, Nashville, TN, USA

## Abstract

Penicillin allergy delabeling may benefit antimicrobial stewardship (AS). Cost of initial penicillin treatments following risk-stratified inpatient delabeling were compared to two hypothetical treatment regimens if delabeling had not occurred: (1) AS-guided and (2) Common Treatment. Penicillin allergy delabeling improved antimicrobial spectrum index, was cost-neutral, and averted unnecessary penicillin desensitizations.

## Background

Although 10%–15% of the United States population carry a penicillin allergy label (PAL), only 1%–5% are confirmed.^1,2^ PALs adversely impact patient care because of use of second-line, less effective, antibiotics, increased side effects, and higher costs.^1^ Using well-validated instruments to identify patients with low-risk PALs that can safely proceed to direct amoxicillin challenge is a successful point-of-care (POC) strategy to enable future penicillin use.^3,4^

Data on the impact of PAL delabeling on antimicrobial stewardship (AS) or antibiotic costs in subsequent treatments is needed. In a patient cohort who received POC risk-stratified PAL delabeling^3,4^ and subsequent treatment with penicillins, we aimed to evaluate the effects of delabeling on antibiotic cost and antimicrobial spectrum index (ASI) of treatment given compared to alternatives they likely would have received without delabeling.^5–7^

## Methods

Forty-five consecutive patients with low-risk PALs (Supplemental Figure 1), primarily cared for by noninfectious-diseases (ID) clinicians, directly challenged with oral amoxicillin and delabeled, who subsequently received penicillin-based treatment between 3/1/2019 and 3/1/2021 were identified from a previously published cohort^3,4^ at Vanderbilt University Medical Center (VUMC). Treatments occurred either immediately following delabeling or in subsequent VUMC inpatient encounters. Age, sex, race, antibiotic indication, regimen, and duration in days were collected. Delabeling performed instead of requested desensitization was also documented.

A panel of 3 ID physicians with AS expertise was convened and asked to assume a counterfactual position in which patients had not been delabeled and provide a consensus opinion on two hypothetical scenarios: 1. Treatment the patient would have received from an AS expert in the presence of their PAL (“AS-Guided”); and 2. Treatment the patient would have received from a non-ID/non-AS-trained physician who might avoid beta-lactams in the presence of a PAL (“Common Treatment”). Results were compared to treatments received (“Delabeled Treatment”). Treatment indications, antibiotics used, and consensus-based alternative treatments for AS-Guided and Common Treatment groups were recorded (Table 1 and Supplemental Table 1).

**Table 1.** Illustrative case examples of delabeled, AS-guided, and common treatment antibiotic regimens including antibiotic duration, median cost, and antimicrobial spectrum index

	Delabeled				AS-Guided				Common Treatment			
	Actually occurred				Predicted to occur if patient NOT delabeled				Predicted to occur if patient NOT delabeled			
**Clinical Scenario Example**	**Abx**	**Days of Tx**	**Total Cost** **(US $)**	**ASI Total**	**Abx**	**Days of Tx**	**Total Cost** **(US $)**	**ASI Total**	**Abx**	**Days of Tx**	**Total Cost** **(US $)**	**ASI Total**
Diabetic wound infection, empiric	TZP	4	136.80	32	VAN	4	72.96	20	VAN	4	72.96	20
					FEP	4	68.40	24	MEM	4	249.24	40
					MTZ	4	12	8				
Total			136.8	8			153.36	13			322.20	15
AMP-susceptible Enterococcus faecalis endocarditis*	AMP	42	594.72	84	AMP	42	594.72	84	AMP	42	594.72	84
					CRO	42	352.80	210	CRO	42	352.80	210
Total			594.72	2			947.52	7			947.52	7
PEN-susceptible Enterococcus faecalis, post-op renal transplant infection*	PEN	14	2811.48	28	PEN	14	2811.48	28	VAN	14	255.36	70
Total			2811.48	2			2811.48	2			255.36	5
Lower respiratory tract infection, immune compromised	AMC	5	6.5	30	AMC	5	6.5	30	LVX	5	2.05	45
Total			6.5	6			6.5	6			2.05	9
Sepsis, empiric	VAN	7	127.68	35	VAN	7	127.68	35	VAN	7	127.68	35
	TZP	7	239.40	56	FEP	7	119.70	42	MEM	7	436.17	70
					MTZ	7	21.00	14				
Total			367.08	13			268.38	13			563.85	15
MSSA infected orthopedic hardware, bacteremia, paraspinal abscess*	NAF	42	2399.04	42	NAF	42	2399.04	42	CFZ	42	963.90	126
Total			2399.04	1			2399.04	1			963.90	3

Includes examples of scenarios from the 45 cases, highlighting that each case, based on available chart documentation regarding allergic response, timing of allergy, and infection severity and the knowledge of the ID/AS experts of the prescribing patterns of their non-ID, non-AS peers, was considered to predict the AS-guided and Common Treatment hypothetical antibiotic prescribing regimens.

Abx, antibiotic; AMP, ampicillin; AMC, amoxicillin-clavulanate; ASI, antimicrobial spectrum index; CFZ, cefazolin; CRO, ceftriaxone; FEP, cefepime; Freq, frequency; LVX, levofloxacin; MEM, meropenem; MSSA, methicillin-sensitive *Staphylococcus aureus*; MTZ, metronidazole; NAF, nafcillin; PEN, penicillin G; post-op, post-operative; Tx, therapy; TZP, piperacillin, tazobactam VAN, vancomycin

Each antibiotic’s median daily hospital acquisition cost across the study period was collected (Supplemental Table 2). For each antibiotic regimen, duration, median daily cost, total cost, and ASI, using previously published values,^5^ were calculated. Dosing intervals were based on existing, infection-dependent, society guidelines, published evidence, and knowledge of institutional practices. Treatment duration for hypothetical AS-Guided and Common Treatment groups was based on available microbiology and prescribing patterns commonly observed in local practice for each group. Disagreements were discussed until consensus was reached. Charges for inpatient procedures and bed costs related to penicillin desensitization were calculated from a previously desensitized patient. The potential for penicillin desensitization was not predicted for AS-Guided or Common Treatment hypothetical groups; therefore, these costs were not included in primary analyses. Median values among the three groups and between groups were analyzed with Kruskal–Wallis test and Wilcoxon ranksum test, respectively. Data were analyzed with Microsoft® Excel (Redmond, Washington) and STATA/MP 16.1 (College Station, Texas).

## Results

The median patient age was 57, and 17/45 (38%) were female. Initial penicillin treatments received after delabeling included 3 amoxicillin (7%), 11 amoxicillin/clavulanate (24%), 1 ampicillin (2%), 6 ampicillin/sulbactam (13%), 1 dicloxacillin (2%), 3 nafcillin (7%), 2 penicillin G (4%), 1 penicillin VK (2%), and 18 piperacillin/tazobactam (40%). Penicillins were given alone in 33 treatments (73%) and combined with other antibiotics in 12 (27%) treatments, most commonly with empiric vancomycin, pending culture and susceptibility results.

Median total antibiotic regimen cost per patient was lower in the AS-Guided group ($43.44; IQR $1.64–$267.48) compared to Delabeled ($61.20; IQR $8.55–$239.40) and Common Treatment ($47.46; IQR $16.80–$322.20) groups but was not statistically significant (*P* = 0.41). Median antibiotic duration was similar across all three groups. Median ASI per patient was lower in AS-Guided (6; IQR 2–8) and Delabeled Treatment groups (6; IQR 6–8) than Common Treatment (8; 5–11), (*P* < 0.01). (Table 2). Compared to Common Treatment, 20 (44%) treatments in the Delabeled Treatment group were less expensive, 3 (7%) were cost-neutral, and 22 (49%) were more expensive.

**Table 2.** Mean and median daily cost, antimicrobial spectrum index, and total cost for delabeled, as-guided, and common treatment groups

	Delabeled Treatment (Group A)	AS-Guided Treatment (Group B)	Common Treatment (Group C)	*P* value^*^	A vs B P value^α^	B vs C P value^α^	A vs C P value^α^
Daily Cost (US $) Median (IQR)	$61.20 ($8.55–$239.40)	$43.44 ($1.64–$267.48)	$47.46 ($16.80–$322.20)	0.41	0.13	0.11	0.87
Antimicrobial Spectrum Index Median (IQR)	6 (6–8)	6 (2–8)	8 (5–11)	<0.01	.05	<0.01	0.08
Duration of Antibiotics Median (IQR)	4 (2–14)	4 (1–14)	4 (1–14)	0.64	0.48	0.42	0.90

* P-values were calculated using Kruskal-Wallis among the three groups.

α Intergroup P-values calculated using Wilcoxon ranksum.

Depending on patient location (non-intensive care unit (ICU) versus ICU) and whether ICU transfer and/or overnight stay was required, an estimated additional $14,000–$70,000 were saved by delabeling PALs in 7 low-risk patients requiring immediate use of penicillin first-line therapy (Supplemental Table 1).

## Discussion

Among 45 patients who received inpatient POC PAL delabeling and subsequent penicillin-based treatment, we observed lower ASI compared to likely alternative treatments in a penicillin-avoidant scenario (i.e., Common Treatment). Risk-stratified PAL assessments, amoxicillin challenge, and delabeling of low-risk penicillin allergies can be performed by a variety of healthcare providers.^3,8^ Trends toward improved ASI in the Delabeled compared to Common Treatment group reflects narrowed, more targeted antibiotics. In the absence of readily available AS expertise, PAL delabeling can potentially increase use of first-line therapy and minimize inappropriately broad antibiotic treatments, reducing antibiotic resistance emergence and antibiotic-associated adverse effects with accumulated benefit over time as patients have future encounters.

The median daily cost of optimal, narrower, first-line treatments like penicillin G was unexpectedly higher than less optimal treatments, highlighting the complexity of demonstrating value of a program that prioritizes quality and safety over cost when those metrics diverge. Because of low costs of many antibiotics, alternative regimens often did not produce dramatic cost differences except the disproportionate cost impacts seen when meropenem, aztreonam, or tobramycin were selected rather than a penicillin. Cefazolin, an equally efficacious alternative beta-lactam, did not necessarily favor penicillin-based treatment on cost alone. However, as noted previously in perioperative settings, PALs promote unnecessary avoidance of cefazolin too,^9^ suggesting some providers would still avoid non-penicillin beta-lactams like cefazolin without delabeling. Although not fully explored in this study, avoidance of unnecessary desensitizations (labor, ICU bed occupancy) likely adds additional savings from risk-stratified PAL delabeling. Regardless, this study supports delabeling as a viable strategy for improving AS which is, at worst, cost-neutral.

This study had limitations. We could not validate the accuracy of the predicted counterfactual treatments, but we attempted to reduce confounding by including three AS experts with significant clinical experience in ID and AS consults to predict treatments based on frequent observation of antibiotic prescribing patterns through usual AS review processes. Future studies could assess median ASI and drug costs given to patients with PALs. Acquisition costs of older intravenous penicillin drugs were unexpectedly expensive during our study period, which may have been an artifact, since IV penicillin G cost peaked during a 2019 shortage before trending down, and ampicillin costs increased 2020–2021 during the COVID-19 pandemic. Nevertheless, median average costs among treatment groups were not significantly different. Additionally, a lower hypothetical total cost of drugs for the Common Treatment group was noted to be primarily associated with use of drugs that, while cheaper, would also provide suboptimal (e.g. vancomycin for methicillin-sensitive *Staphylococcus aureus* infection) or overly broad treatment (e.g. meropenem for empiric sepsis treatment without prior evidence of multi-drug resistance). Finally, because alternative regimens were hypothetical, we could not assess costs of unobserved treatment failures or adverse events that may have resulted from use of less effective second-line therapy with broader antimicrobial activity, another hidden, significant cost of penicillin avoidance.

## Conclusions

Risk-stratified PAL delabeling is an effective tool for optimizing appropriate antibiotic choice. This approach is cost-neutral for antibiotic costs and likely cost-saving when considering avoidance of additional healthcare costs including adverse drug events and expensive penicillin desensitizations.

## Supporting information

Staub et al. supplementary material Staub et al. supplementary material

## Supplementary material

For supplementary material accompanying this paper visit http://doi.org/10.1017/ash.2024.421. click here to view supplementary material
