# Supplementary material for: Impacts of risk-stratified inpatient penicillin allergy label delabeling on subsequent antimicrobial spectrum index and costs
Source: Antimicrob Steward Healthc Epidemiol. 2024 Oct 3;4(1):e160. doi: 10.1017/ash.2024.421 (PMC11450671; doi:10.1017/ash.2024.421)
Supplement: Staub et al. supplementary material [file S2732494X24004212sup001.docx]

**Table 1: Delabeled, AS-Guided, and Common Treatment Antibiotic Regimens, Duration, Median Cost, and Antimicrobial Spectrum Index**

|  | **Delabeled** | | | | **AS-Guided** | | | | **Common Treatment** | | | |
| --- | --- | --- | --- | --- | --- | --- | --- | --- | --- | --- | --- | --- |
| **Clinical Scenario** | **Abx** | **Days of Tx** | **Total Cost**  **(US $)** | **ASI Total** | **Abx** | **Days of Tx** | **Total Cost**  **(US $)** | **ASI Total** | **Abx** | **Days of Tx** | **Total Cost**  **(US $)** | **ASI Total** |
| Diabetic wound infection, empiric | TZP | 4 | 136.80 | 32 | VAN | 4 | 72.96 | 20 | VAN | 4 | 72.96 | 20 |
|  |  |  |  |  | FEP | 4 | 68.40 | 24 | MEM | 4 | 249.24 | 40 |
|  |  |  |  |  | MTZ | 4 | 12 | 8 |  |  |  |  |
| Total |  |  | 136.8 | 8 |  |  | 153.36 | 13 |  |  | 322.20 | 15 |
| Peri-op ppx, tunneled line | SAM | 1 | 5.1 | 6 | None |  |  |  | None |  |  |  |
| Total |  |  | 5.1 | 6 |  |  |  | 0 |  |  |  | 0 |
| AMP-susceptible Enterococcus faecalis endocarditis* | AMP | 42 | 594.72 | 84 | AMP | 42 | 594.72 | 84 | AMP | 42 | 594.72 | 84 |
|  |  |  |  |  | CRO | 42 | 352.80 | 210 | CRO | 42 | 352.80 | 210 |
| Total |  |  | 594.72 | 2 |  |  | 947.52 | 7 |  |  | 947.52 | 7 |
| Febrile neutropenia | TZP | 7 | 239.40 | 56 | TZP | 7 | 239.40 | 56 | ATM | 14 | 1229.76 | 42 |
| Total |  |  | 239.40 | 8 |  |  | 239.40 | 8 |  |  | 1229.76 | 3 |
| Febrile, immune suppressed | TZP | 2 | 68.40 | 16 | FEP (1) | 1 | 17.10 | 6 | FEP (1) | 1 | 17.10 | 6 |
|  |  |  |  |  | CRO (2) | 1 | 8.40 | 5 | CRO (2) | 1 | 8.40 | 5 |
| Total |  |  | 68.40 | 8 |  |  | 25.50 | 5.5 |  |  | 25.50 | 5.5 |
| MSSA hand infection | DCX | 14 | 36.96 | 14 | DOX | 5 | 15.65 | 25 | LEX | 10 | 12.00 | 20 |
| Total |  |  | 36.96 | 1 |  |  | 15.65 | 5 |  |  | 12.00 | 2 |
| PEN-susceptible Enterococcus faecalis, post- op renal transplant infection* | PEN | 14 | 2811.48 | 28 | PEN | 14 | 2811.48 | 28 | VAN | 14 | 255.36 | 70 |
| Total |  |  | 2811.48 | 2 |  |  | 2811.48 | 2 |  |  | 255.36 | 5 |
| Lower respiratory tract infection | AZM | 2 | 10.30 | 8 | None |  |  |  | AZM (1) | 2 | 10.30 | 8 |
|  | AMC | 4 | 5.20 | 24 |  |  |  |  | CRO (1) | 2 | 16.80 | 10 |
|  |  |  |  |  |  |  |  |  | LVX (2) | 2 | 0.82 | 18 |
| Total |  |  | 15.50 | 8 |  |  |  | 0 |  |  | 27.92 | 9 |
| Peri-op ppx, nephrostomy | SAM | 1 | 5.1 | 6 | FEP | 1 | 5.70 | 6 | MEM | 1 | 20.77 | 10 |
| Total |  |  | 5.1 | 6 |  |  | 5.70 | 6 |  |  | 20.77 | 10 |
| Lower respiratory tract infection, immune compromised | AMC | 5 | 6.5 | 30 | AMC | 5 | 6.5 | 30 | LVX | 5 | 2.05 | 45 |
| Total |  |  | 6.5 | 6 |  |  | 6.5 | 6 |  |  | 2.05 | 9 |
| Lower respiratory tract infection, sepsis | TZP(1,2) | 5 | 171 | 40 | TZP | 4 | 136.80 | 32 | MEM | 14 | 872.34 | 140 |
|  | LVX(2) | 1 | 0.41 | 9 |  |  |  |  | VAN | 14 | 255.36 | 70 |
| Total |  |  | 171.41 | 9.8 |  |  | 136.80 | 8 |  |  | 1127.7 | 15 |
| Sacral osteomyelitis | VAN (1) | 13 | 237.12 | 65 | TZP | 42 | 1436.40 | 336 | VAN | 13 | 237.12 | 65 |
|  | TZP (1,2) | 42 | 1436.40 | 336 | SXT | 42 | 21.00 | 168 | MEM | 42 | 2617.02 | 420 |
|  | SXT (2) | 29 | 14.5 | 116 |  |  |  |  |  |  |  |  |
| Total |  |  | 1688.02 | 12.3 |  |  | 1457.40 | 12 |  |  | 2854.14 | 11.5 |
| Peri-op ppx, liver transplant | SAM | 2 | 61.20 | 12 | SAM | 2 | 61.20 | 12 | FEP | 2 | 34.2 | 12 |
|  |  |  |  |  |  |  |  |  | MTZ | 2 | 6.00 | 4 |
| Total |  |  | 61.20 | 6 |  |  | 61.20 | 6 |  |  | 40.20 | 8 |
| Hospital acquired pneumonia | VAN | 1 | 18.24 | 5 | VAN | 1 | 18.24 | 5 | VAN | 1 | 18.24 | 5 |
|  | AMC | 3 | 3.90 | 18 | CRO | 3 | 25.20 | 15 | CRO | 1 | 8.40 | 5 |
|  |  |  |  |  |  |  |  |  | LVX (2) | 2 | 0.82 | 18 |
| Total |  |  | 22.14 | 7.67 |  |  | 43.44 | 6.67 |  |  | 27.46 | 9.33 |
| Sepsis, empiric | VAN | 7 | 127.68 | 35 | VAN | 7 | 127.68 | 35 | VAN | 7 | 127.68 | 35 |
|  | TZP | 7 | 239.40 | 56 | FEP | 7 | 119.70 | 42 | MEM | 7 | 436.17 | 70 |
|  |  |  |  |  | MTZ | 7 | 21.00 | 14 |  |  |  |  |
| Total |  |  | 367.08 | 13 |  |  | 268.38 | 13 |  |  | 563.85 | 15 |
| Sepsis, empiric | VAN | 1 | 18.24 | 5 | None |  |  |  | VAN | 1 | 18.24 | 5 |
|  | TZP | 1 | 34.20 | 8 |  |  |  |  | FEP | 1 | 17.10 | 6 |
| Total |  |  | 52.44 | 13 |  |  |  | 0 |  |  | 35.34 | 11 |
| Diabetic wound, empiric | VAN | 2 | 36.48 | 10 | VAN | 3 | 54.72 | 15 | VAN | 3 | 54.72 | 15 |
|  | TZP | 3 | 102.60 | 24 | FEP | 3 | 51.3 | 18 | FEP | 3 | 51.3 | 18 |
|  |  |  |  |  | MTZ | 3 | 9.00 | 6 | MTZ | 3 | 9.00 | 6 |
| Total |  |  | 139.08 | 11.3 |  |  | 115.02 | 13 |  |  | 115.02 | 13 |
| Hematopoietic stem cell transplant ppx | PCV | 4 | 2.72 | 8 | LVX | 4 | 1.64 | 36 | LVX | 4 | 1.64 | 36 |
| Total |  |  | 2.72 | 2 |  |  | 1.64 | 9 |  |  | 1.64 | 9 |
| Post-op cellulitis | VAN (1) | 3 | 54.72 | 15 | VAN (1) | 3 | 54.72 | 15 | VAN (1) | 3 | 54.72 | 15 |
|  | TZP (1) | 3 | 102.60 | 24 | CAZ (1) | 3 | 62.82 | 12 | CRO (1) | 3 | 25.20 | 15 |
|  | AMC (2) | 1 | 1.30 | 6 | LEX (2) | 1 | 1.20 | 2 | LEX (2) | 1 | 1.20 | 2 |
| Total |  |  | 158.62 | 11.3 |  |  | 118.74 | 7.25 |  |  | 81.12 | 8 |
| Aspiration pneumonia | AMC | 4 | 6.08 | 24 | None |  |  |  | CRO | 4 | 33.60 | 20 |
|  |  |  |  |  |  |  |  |  | MTZ | 4 | 12.00 | 8 |
| Total |  |  | 6.08 | 6 |  |  |  | 0 |  |  | 45.6 | 7 |
| Aspiration pneumonia, ICU | SAM | 1 | 30.6 | 6 | VAN | 1 | 18.24 | 5 | VAN | 1 | 18.24 | 5 |
|  |  |  |  |  | FEP | 1 | 17.10 | 6 | FEP | 1 | 17.10 | 6 |
| Total |  |  | 30.6 | 6 |  |  | 35.34 | 11 |  |  | 35.34 | 11 |
| MSSA infected orthopedic hardware, bacteremia, paraspinal abscess* | NAF | 42 | 2399.04 | 42 | NAF | 42 | 2399.04 | 42 | CFZ | 42 | 963.90 | 126 |
| Total |  |  | 2399.04 | 1 |  |  | 2399.04 | 1 |  |  | 963.90 | 3 |
| Sepsis, empiric | VAN | 2 | 36.48 | 10 | None |  |  |  | VAN | 2 | 36.48 | 10 |
|  | TZP | 2 | 68.40 | 16 |  |  |  |  | FEP | 2 | 34.20 | 12 |
|  | AZM | 2 | 10.3 | 8 |  |  |  |  |  |  |  |  |
| Total |  |  | 115.18 | 17 |  |  |  | 0 |  |  | 70.68 | 11 |
| Peri-op ppx, NSSTI I&D | TZP | 1 | 8.55 | 8 | CRO | 1 | 4.20 | 5 | ATM | 1 | 29.28 | 3 |
| Total |  |  | 8.55 | 8 |  |  | 4.20 | 5 |  |  | 29.28 | 3 |
| Lower respiratory tract infection, empiric | VAN | 1 | 18.24 | 5 | None |  |  |  | TZP | 1 | 34.20 | 8 |
|  | TZP | 1 | 34.20 | 8 |  |  |  |  |  |  |  |  |
| Total |  |  | 52.44 | 13 |  |  |  | 0 |  |  | 34.20 | 8 |
| Epiglottitis, outpatient treatment completion | AMX | 10 | 1.60 | 20 | AMX | 10 | 1.60 | 20 | CDR | 10 | 13.2 | 30 |
| Total |  |  | 1.60 | 2 |  |  | 1.60 | 2 |  |  | 13.2 | 3 |
| MSSA osteomyelitis, epidural abscess* | NAF | 42 | 2399.04 | 42 | NAF | 42 | 2399.04 | 42 | CFZ | 42 | 963.90 | 126 |
| Total |  |  | 2399.04 | 1 |  |  | 2399.04 | 1 |  |  | 963.90 | 3 |
| Superficial ostomy infection | AMC | 1 | 1.30 | 6 | None |  |  |  | AMC | 1 | 1.30 | 6 |
| Total |  |  | 1.30 | 6 |  |  |  | 0 |  |  | 1.30 | 6 |
| Syphilis* | BPG | 3 | 100.41 | 6 | BPG | 3 | 100.41 | 6 | DOX | 21 | 47.46 | 105 |
| Total |  |  | 100.41 | 2 |  |  | 100.41 | 2 |  |  | 47.46 | 5 |
| Pseudomonas aeruginosa pneumonia, cystic fibrosis | TZP | 13 | 444.60 | 104 | CAZ | 13 | 272.22 | 52 | CAZ | 13 | 272.22 | 52 |
|  |  |  |  |  | TOB | 13 | 56.43 | 65 | TOB | 13 | 56.43 | 65 |
| Total |  |  | 444.60 | 8 |  |  | 328.65 | 9 |  |  | 328.65 | 9 |
| Possible cholecystitis, empiric | TZP | 1 | 8.55 | 8 | FEP | 1 | 5.70 | 6 | FEP | 1 | 5.70 | 6 |
|  |  |  |  |  | MTZ | 1 | 1.00 | 2 | MTZ | 1 | 100 | 2 |
| Total |  |  | 8.55 | 8 |  |  | 6.70 | 8 |  |  | 6.70 | 8 |
| Aspiration pneumonia | AMC(2) | 1 | 1.30 | 6 | SAM | 3 | 91.8 | 18 | FEP | 3 | 51.30 | 18 |
|  | SAM (1) | 2 | 61.2 | 12 |  |  |  |  | MTZ | 3 | 9.00 | 6 |
|  |  |  |  |  |  |  |  |  | AZM | 3 | 15.45 | 12 |
| Total |  |  | 62.50 | 6 |  |  | 91.8 | 6 |  |  | 75.75 | 12 |
| Febrile neutropenia, empiric | TZP | 1 | 34.20 | 8 | LVX | 1 | 0.41 | 9 | MEM | 1 | 62.31 | 10 |
| Total |  |  | 34.20 | 8 |  |  | 0.41 | 9 |  |  | 62.31 | 10 |
| Diverticulitis, completion | AMC | 1 | 1.30 | 6 | CRO | 1 | 8.40 | 5 | CRO | 1 | 8.40 | 5 |
|  |  |  |  |  | MTZ | 1 | 3.00 | 2 | MTZ | 1 | 3.00 | 2 |
| Total |  |  | 1.30 | 6 |  |  | 11.40 | 7 |  |  | 11.40 | 7 |
| Streptococcal pneumoniae meningitis* | PEN | 14 | 2811.48 | 28 | PEN | 14 | 2811.48 | 28 | CRO | 14 | 117.60 | 70 |
| Total |  |  | 2811.48 | 2 |  |  | 2811.48 | 2 |  |  | 117.60 | 5 |
| Empyema, treatment completion | AMC | 5 | 6.5 | 30 | SXT | 14 | 7.00 | 56 | LVX | 14 | 5.74 | 126 |
| Total |  |  | 6.5 | 6 |  |  | 7.00 | 4 |  |  | 5.74 | 9 |
| Dental ppx, renal transplant | AMX | 1 | 0.08 | 2 | None |  |  |  | AMX | 1 | 0.08 | 2 |
| Total |  |  | 0.08 | 2 |  |  |  | 0 |  |  | 0.08 | 2 |
| Febrile neutropenia, empiric | TZP | 14 | 478.80 | 112 | CAZ | 14 | 293.16 | 56 | MEM | 14 | 872.34 | 140 |
| Total |  |  | 478.80 | 8 |  |  | 293.16 | 4 |  |  | 872.34 | 10 |
| Cholangitis* | SAM | 14 | 428.40 | 84 | SAM | 14 | 428.40 | 84 | VAN | 14 | 255.36 | 70 |
|  |  |  |  |  |  |  |  |  | CRO | 14 | 117.60 | 70 |
|  |  |  |  |  |  |  |  |  | MTZ | 14 | 42.00 | 28 |
| Total |  |  | 428.40 | 6 |  |  | 428.40 | 6 |  |  | 414.96 | 12 |
| MSSA bacteremia | NAF | 28 | 1599.36 | 28 | FEP | 28 | 478.80 | 84 | VAN | 28 | 510.72 | 140 |
|  | CIP | 28 | 10.64 | 224 |  |  |  |  | FEP | 28 | 478.80 | 168 |
|  | MTZ | 28 | 84.00 | 56 |  |  |  |  | MTZ | 28 | 84.00 | 56 |
| Total |  |  | 1694 | 11 |  |  | 478.80 | 3 |  |  | 1073.52 | 13 |
| Pulmonary abscess | AMC | 42 | 54.6 | 252 | AMC | 42 | 54.6 | 252 | LVX | 42 | 17.22 | 378 |
|  |  |  |  |  |  |  |  |  | MTZ | 42 | 126 | 84 |
| Total |  |  | 54.6 | 6 |  |  | 54.6 | 6 |  |  | 143.22 | 11 |
| Spontaneous bacterial peritonitis | TZP | 2 | 68.40 | 16 | CRO | 2 | 16.80 | 10 | CRO | 2 | 16.80 | 10 |
| Total |  |  | 68.40 | 8 |  |  | 16.80 | 5 |  |  | 16.80 | 5 |
| AMX susceptible Enterococcus faecalis UTI | AMX | 5 | 0.80 | 10 | AMX | 5 | 0.80 | 10 | AMC | 7 | 9.10 | 42 |
| Total |  |  | 0.80 | 2 |  |  | 0.80 | 2 |  |  | 9.10 | 6 |
| Mandibular osteomyelitis | AMC | 42 | 54.6 | 252 | AMC | 42 | 54.6 | 252 | AMC | 42 | 54.6 | 252 |
| Total |  |  | 54.6 | 6 |  |  | 54.6 | 6 |  |  | 54.6 | 6 |
| Sepsis from a urinary source, empiric | TZP | 4 | 136.80 | 32 | VAN | 1 | 18.24 | 5 | VAN | 4 | 72.96 | 20 |
|  |  |  |  |  | MEM | 4 | 249.24 | 40 | MEM | 4 | 249.24 | 40 |
| Total |  |  | 136.80 | 8 |  |  | 267.48 | 11.3 |  |  | 322.20 | 15 |

Abbreviations: Abx- antibiotic; AMP- ampicillin; AMC- amoxicillin-clavulanate; AMX-amoxicillin; ATM-aztreonam; AZM-azithromycin; BPG- benzathine penicillin; CAZ-ceftazidime; CDR- cefdinir; CFZ- cefazolin; CIP-ciprofloxacin; CRO- ceftriaxone; DCX-dicloxacillin; DOX-doxycycline; FEP-cefepime; Freq- frequency; LEX-cephalexin; LVX- levofloxacin; MEM-meropenem; MTZ-metronidazole; NAF-nafcillin; NSSTI- necrotizing skin and soft tissue infection; PEN- penicillin G; post-op- post-operative; ppx- prophylaxis; SAM-ampicillin-sulbactam; SXT-trimethoprim-sulfamethoxazole; TOB-tobramycin; Tx- therapy; TZP- piperacillin-tazobactam VAN-vancomycin

*Desensitization avoided

When parentheses are included by drug names, it indicates there was a regimen change and the number is which regimen the drug was included in.

Total row data reports either the Total Cost or for Total ASI, the sum of all ASI divided by days of therapy.

**Supplemental Figure 1: Criteria for Low-Risk Penicillin Allergy**

1. Urticaria alone, >5 years ago
2. Self-limited cutaneous rash, no organ involvement or signs of severe, delayed rash
3. Gastrointestinal symptoms alone
4. Remote childhood reaction, limited details
5. Family history penicillin allergy alone
6. Avoidance from fear of allergy alone
7. Known penicillin tolerance since original reaction
8. Other non-allergy symptoms

**Direct Oral Challenge with Amoxicillin**

*****Criteria from Koo G, et al. J Allergy Clin Immunol Pract, 2023.

**Supplemental Table 2: Antibiotic Names, Abbreviations, Standard Daily Doses, Mean Cost per Dose, and ASI per Day Used in this Study**

| Antibiotic | Abbreviation | Standard Daily Doses | Mean Cost per Dose (US$) ^a, b^ | Daily Antimicrobial Stewardship Index Value ^c^ |
| --- | --- | --- | --- | --- |
| Amoxicillin | AMX | 2 | 0.11 | 2 |
| Amoxicillin/Clavulanate | AMC | 2 | 0.76 | 6 |
| Ampicillin | AMP | 6 | 7.43 | 2 |
| Ampicillin/Sulbactam | SAM | 6 | 5.10 | 6 |
| Azithromycin | AZM | 1 | 5.42 | 4 |
| Aztreonam | ATM | 3 | 41.89 | 3 |
| Cefazolin | CFZ | 3 | 8.15 | 3 |
| Cefepime | FEP | 3 | 7.14 | 6 |
| Cefdinir | CDR | 2 | 0.71 | 3 |
| Ceftazidime | CAZ | 3 | 8.15 | 4 |
| Ceftriaxone | CRO | 2 | 4.80 | 5 |
| Cephalexin | LEX | 4 | 0.34 | 2 |
| Ciprofloxacin | CIP | 2 | 1.82 | 8 |
| Dicloxacillin | DCX | 4 | 20.78 | 1 |
| Doxycycline | DOX | 2 | 7.60 | 5 |
| Levofloxacin | LVX | 1 | 2.94 | 9 |
| Meropenem | MEM | 3 | 20.15 | 10 |
| Metronidazole | MTZ | 3 | 1.63 | 2 |
| Nafcillin | NAF | 6 | 14.89 | 1 |
| Penicillin G | PEN | 6 | 32.13 | 2 |
| Benzathine Penicillin G | BPG | 1* | 31.13 | 2 |
| Penicillin VK | PCV | 4 | 0.13 | 2 |
| Piperacillin/Tazobactam | TZP | 4 | 8.73 | 8 |
| Sulfamethoxazole/Trimethoprim | SXT | 2 | 1.97 | 4 |
| Tobramycin | TOB | 3 | 66.90 | 5 |
| Vancomycin | VAN | 2 | 9.02 | 5 |

^a^ Cost only included hospital acquisition cost, obtained from our institution’s pharmacy department for the entire study period, from which the median daily cost was calculated.

^b^ The hospital acquisition costs did not differentiate between formulation, but rather was an aggregate of all formulations used during the study period.

^b^ Used modified Antimicrobial Spectrum Index from Gerber, et al. *Infect Control Hosp Epidemiol,* 2017.

*weekly
